# Editorial: Sub-molecular mechanism of genetic epilepsy

**DOI:** 10.3389/fnmol.2022.958747

**Published:** 2022-07-26

**Authors:** Wei-Ping Liao, Qian Chen, Yu-Wu Jiang, Sheng Luo, Xiao-Rong Liu

**Affiliations:** ^1^Department of Neurology, Institute of Neuroscience, The Second Affiliated Hospital of Guangzhou Medical University, Guangzhou, China; ^2^Key Laboratory of Neurogenetics and Channelopathies of Guangdong Province, Ministry of Education of China, Guangzhou, China; ^3^Department of Brain and Cognitive Sciences, McGovern Institute for Brain Research, Massachusetts Institute of Technology, Cambridge, MA, United States; ^4^Department of Pediatrics, Peking University First Hospital, Peking, China

**Keywords:** genetic epilepsy, sub-molecular mechanism, genotype, phenotypic variation, functional domain

Proteins are the essential functional molecule in living organisms. A protein is featured by finely structured domains, which are encoded by genetic sequences. Variants in a gene potentially result in functional alteration of the protein and thus cause diseases. From the perspective of molecular structure, the different domains of a protein play distinct roles. Variants of different locations are thus potentially associated with various damaging effects and subsequently lead to phenotypical variation. Similarly, the bio-physical feature of the substituted amino acids caused by variants is also a determinant of the damaging effect. Such submolecular implication would be a critical factor in determining the pathogenicity of variants.

More than 4,000 genes have been associated with human diseases (https://omim.org/). A gene could be associated with a distinct phenotype, but in the majority of circumstances, it is associated with a spectrum of phenotypes that are varied in severity or other aspects, for which the underlying mechanism is mostly unknown. Previously, sub-molecular implications have been demonstrated to be a determinant of phenotypical variation in several molecules, such as sodium channel Nav1.1 (*SCN1A*) (Meng et al., 2015; Tang et al., 2020), tyrosine 3-monooxygenase/tryptophan 5-monooxygenase activation protein gamma (*YWHAG*) (Ye et al., 2021), and disheveled Egl-10 and pleckstrin domain-containing protein 5 (*DEPDC5*) (Liu et al., 2020).

The present Research Topic is dedicated to exploring the *Sub-molecular Mechanism of Genetic Epilepsy* and providing novel perspectives to understanding of the mechanisms underlying the phenotypical variation of genetic diseases. The sub-molecular implications of genetic variants are disclosed in terms of six aspects in this collection.

## Functional domain and sub-molecular implication

Shu et al. identified four *de novo* missense *MAST3* variants from four patients with heterotypic neurodevelopmental disorders (NDD), including two with intellectual disability (ID) and epilepsy and two with ID and autism spectrum disorders (ASD). They created a *mast3a/b* knockout zebrafish model and observed abnormal morphology of the central nervous system. The results support the possibility that *MAST3* is a novel gene associated with NDD. Additionally, ASD-related missense variants presented higher frequencies in the DUF domain, while epilepsy-related variants exhibited higher frequencies in the STK domain, suggesting a sub-regional effect.

Bian et al. identified six hemizygous missense *SHROOM4* variants from six cases with epilepsy without ID. The *SHROOM4* gene was previously reported in patients with the Stocco dos Santos type X-linked syndromic intellectual developmental disorder (SDSX) (Hagens et al., 2006). The SDSX-related variants reported were mostly destructive or duplicative variants, while the epilepsy-related variants were all missense variants located around the N-terminal PDZ domain and the C-terminal ASD2 domain, indicating a molecular sub-regional effect.

Zou et al. identified five hemizygous missense *AFF2* mutations from five males with partial epilepsy and antecedent febrile seizures without intellectual disability or other developmental abnormalities. The *AFF2* gene is previously reported to be associated with X-linked intellectual developmental disorder 109 and ASD (Stettner et al., 2011). The ID-associated *AFF2* mutations were mostly genomic rearrangements and CCG repeats expansion mutations that caused gene silencing, whereas the mutations associated with epilepsy or ASD were all missense, suggesting the correlation between the phenotype and variant type. Furthermore, epilepsy-relative *AFF2* variants fell into the regions from N-terminal to the nuclear localization signal 1 (NLS1), while ASD-associated missense mutations were located in the regions from NLS1 to C-terminal, suggesting a sub-region effect.

Li et al. identified 12 *CACNA1A* variants and analyzed the correlation between genotype and phenotype. This study suggested that *CACNA1A* mutations were potentially associated with a spectrum of epileptic phenotypes, ranging from mild absence epilepsy to severe developmental and epileptic encephalopathy (DEE). Further analysis revealed that: 1) episodic ataxia type 2 had a tendency of higher frequency of null variants than epilepsies; 2) the missense variants in severe epileptic phenotypes were more frequently located in the pore region than those in milder epileptic phenotypes; and 3) *de novo* variants in epilepsy were more frequently associated with ID.

A functional molecular unit may be a single molecule encoded by a gene or a molecular complex consisting of several proteins encoded by different genes. Previously, the A subunit of the V1 domain of V-ATPase encoded by *ATP6V1A* was associated with DEE-93 (Fassio et al., 2018). The *ATP6V0C* gene encoded the C subunit of the V0 domain of V-ATPase. Tian Y. et al. identified *ATP6V0C* as a novel causative gene for febrile seizures (FS) and epilepsy with febrile seizures plus (EFS+), providing a novel perspective of the functional domain and sub-molecular implication.

## Substituted amino acid and sub-molecular implication

Su et al. focus on the relationship between the nature of substituted amino acids and functional alteration. They identified a novel missense *SCN1A* variant (c.4868A>C/p.E1613A) in the extracellular S3-S4 loop of Nav1.1 from two cases with epilepsy. Functional studies were conducted on six mutants with all possible substitutions in the residue E1623. This study suggested the critical role of the S3–S4 loop in sodium channel function. Analysis of the correlation between the residue properties and electrophysiological alterations demonstrated that hydrophilicity of the side-chain at E1623 was potentially a crucial contributor to voltage-dependent kinetics, whereas the contributor to the channel conductance, which is closely associated with epileptogenesis, remains undetermined.

## Isoforms and sub-molecular implication

Zhou et al. identified 12 *ADGRV1* variants in nine unrelated cases with FS or EFS+. Previously, the *ADGRV1* gene was reported to be associated with Usher syndrome (Weston et al., 2004). The authors reviewed all *ADGRV1* variants and analyzed the genotype-phenotype correlations of the *ADGRV1* variants in epilepsy and audio-visual disorders. This study showed that the epilepsy-related variants were monoallelic, missense, and mainly located at the CalX-β domain. Furthermore, the epilepsy-related variants mostly affected isoforms VLGR1b/1c, while the audio-visual-related variants were mainly affected isoforms VLGR1a, suggesting a potential role of isoform in phenotype variation.

## Monoallelic and biallelic variants

Wang et al. identified eight pairs of compound heterozygous missense *PKD1* variants in eight patients with FS or EFS+. Dominant *PKD1* variants were reported to be associated with polycystic kidney disease (Roelfsema et al., 1997). Further analysis suggested that monoallelic mutations with haploinsufficiency of *PKD1* were potentially associated with kidney disease, compound heterozygotes with superimposed effects of two missense mutations were associated with epilepsy; whereas the homozygotes with complete loss of *PKD1* would be embryonically lethal, suggesting monoallelic variant/biallelic variant was potentially a factor in determining phenotypical variation.

## Locations of the two variants in a pair of compound heterozygous variant

Luo et al. identified six pairs of compound heterozygous missense *LAMA5* variants in six unrelated infants with partial epilepsy and spasms. This article identified *LAMA5* as a novel potential epilepsy gene. Interestingly, among the biallelic variants in cases with mild phenotype, two variants of each pair were located in different structural domains or domains/links, whereas in the cases with spasms (the severer phenotype), the biallelic variants were constituted by two variants in the identical functional domains, potentially suggesting a novel perspective on sub-molecular mechanisms.

## Specific genotype and sub-molecular implication

Wang et al. identified six in-frame deletion variants in *SCN1A* and performed further functional analysis. The experiments showed the complete loss of function caused by the six in-frame deletion variants, emphasizing the damaging effect of in-frame deletion variants.

Truncated C-terminal region *MN1* variants caused *MN1* C-terminal truncation (MCTT) syndrome (Mak et al., 2020). Tian Q. et al. reported a novel C-terminal null *MN1* variant (p.L1245fs) in a patient with mild developmental delay without structural brain abnormalities, expanding the clinical and genetic spectrum of MCTT.

In conclusion, this Research Topic provides novel perspectives on the mechanisms underlying phenotypical variation. Phenotypical heterogeneity is common in genetic disorders. We advocate further studies on the submolecular mechanism of genetic diseases beyond epilepsy, toward setting a submolecular stage of medicine.

## Author contributions

All authors listed have made a substantial, direct, and intellectual contribution to the work and approved it for publication.

## Conflict of interest

The authors declare that the research was conducted in the absence of any commercial or financial relationships that could be construed as a potential conflict of interest.

## Publisher's note

All claims expressed in this article are solely those of the authors and do not necessarily represent those of their affiliated organizations, or those of the publisher, the editors and the reviewers. Any product that may be evaluated in this article, or claim that may be made by its manufacturer, is not guaranteed or endorsed by the publisher.
